# Experience with Laparoscopic Surgery for Rectal Obstruction Caused by Intestinal Endometriosis with a Frozen Pelvis: A Case Report

**DOI:** 10.70352/scrj.cr.24-0148

**Published:** 2025-05-13

**Authors:** Hisashi Ro, Yuki Tsuchiya, Ryoichi Tsukamoto, Kumpei Honjo, Masaya Kawai, Shun Ishiyama, Kiichi Sugimoto, Makoto Takahashi, Mari Kitade, Harumi Saeki, Takashi Yao, Kazuhiro Sakamoto

**Affiliations:** 1 Department of Coloproctological Surgery, Juntendo University Faculty of Medicine, Juntendo University, Bunkyo, Tokyo, Japan; 2 Department of Obstetrics and Gynecology, Juntendo University Faculty of Medicine, Juntendo University, Bunkyo, Tokyo, Japan; 3 Department of Human Pathology, Juntendo University Faculty of Medicine, Juntendo University, Bunkyo, Tokyo, Japan; 4 Department of Human Pathology, Juntendo University Graduate School of Medicine, Juntendo University, Bunkyo, Tokyo, Japan

**Keywords:** intestinal endometriosis, obstruction, laparoscopic surgery, gynecologists, frozen pelvis, collaboration

## Abstract

**INTRODUCTION:**

While intestinal obstruction is common, intestinal endometriosis is relatively rare, making its etiology still poorly understood. We report a case of rectal obstruction caused by intestinal endometriosis with a frozen pelvis, treated with laparoscopy in collaboration with gynecologists.

**CASE PRESENTATION:**

A 39-year-old female patient was diagnosed with rectal obstruction resulting from endometriosis with a frozen pelvis by her previous physician and subsequently treated with a transverse colon stoma for rectal obstruction and hormonal therapy for endometriosis. Unfortunately, her condition did not improve after hormonal therapy at our hospital; hence, laparoscopic low anterior resection and pelvic unclogging were performed. Regarding the extent of intestinal dissection, the rectal dissection was performed by dissecting to the point where no tissue changes were observed on the serosal side. Pathological findings revealed endometrial gland-like ducts and intimal stromal hyperplasia spanning from the serosa to the submucosa of the rectum. Her postoperative course was uneventful, leading to her discharge on postoperative day 15. The stoma was closed postoperatively, and gastrointestinal symptoms such as bowel obstruction and bleeding did not recur.

**CONCLUSIONS:**

The extent of intestinal endometriosis preoperatively is difficult to determine accurately. Identifying the extent of bowel resection has a significant impact on the patient’s postoperative activities of daily living. Thus, carefully observing the lesion intraoperatively and resecting it at a sufficient distance from the occluding lesion are advisable.

## Abbreviations

DST
: double stapling technique
GnRH
: gonadotropin-releasing hormone

## INTRODUCTION

Endometriosis is a disease characterized by the ectopic proliferation of endometrial tissue, affecting approximately 10% of adult women.^1)^ In particular, intestinal endometriosis reportedly accounts for 5%–37% of all endometriosis cases.^2)^ Its clinical manifestations include abdominal pain, melena, dysesthesia, and defecation difficulty, which appear to fluctuate in severity according to the menstrual cycle.^3,4)^ Surgery is the treatment of choice for patients with recurrent symptoms or unresponsiveness to hormonal therapy. Intestinal endometriosis extends beyond the obstructive lesion. The extent of resection must be determined to maintain the postoperative quality of life. Herein, we report a case of rectal obstruction caused by intestinal endometriosis with a frozen pelvis, treated laparoscopically, with no gastrointestinal symptoms postoperatively.

## CASE PRESENTATION

**Fig. 1:**
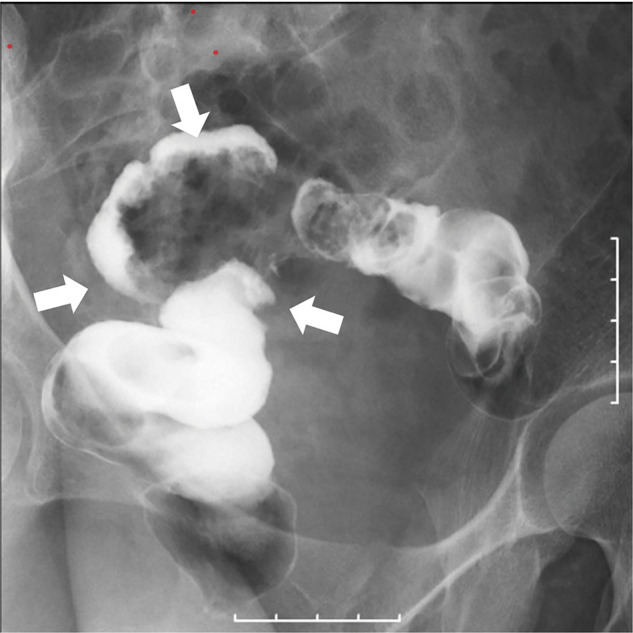
Obstruction (arrow) was observed from the sigmoid to the rectum. The wall of the oral side of the intestine was irregular and sclerotic.

**Fig. 2:**
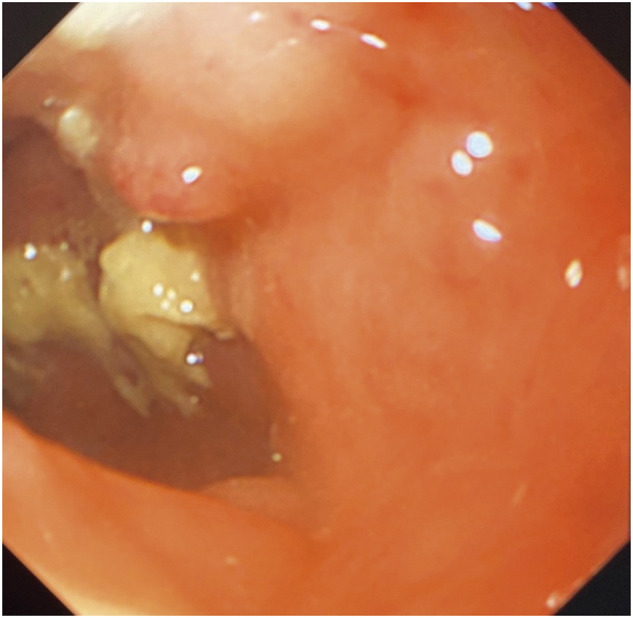
Radiographic colonoscopy showed an obstruction at the rectum that prevented passage of the scope. The site of obstruction on the anal side was 10 cm from the anal verge.

**Fig. 3:**
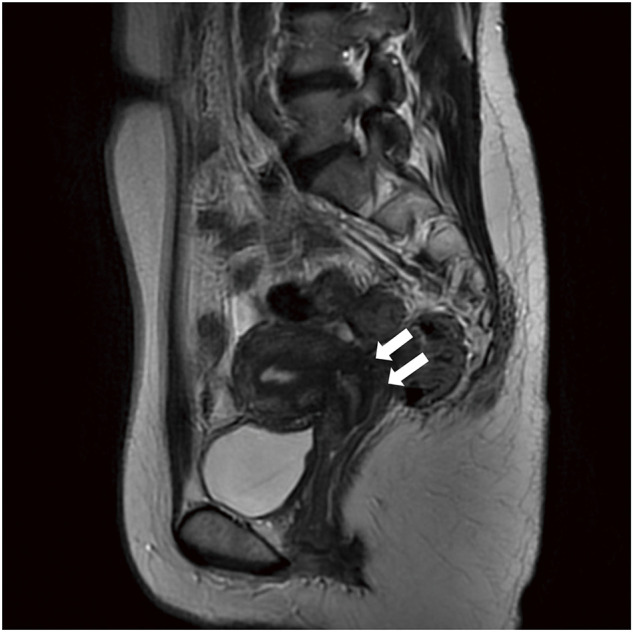
T2 image shows that a low signal mass was observed in the posterior vaginal vault and the anterior rectal wall (arrow).

A 39-year-old female presented to a previous hospital, mainly complaining of abdominal pain. She was diagnosed with endometriosis in her teens which was treated with hormonal therapy but was recently stopped because of self-decision. In another hospital, she was diagnosed with rectal obstruction caused by intestinal endometriosis. Hence, she was urgently hospitalized, undergoing transverse colostomy via an open surgery. Hormonal therapy (GnRH), as well as Dienogest, was then administered. However, her condition did not improve. Fourteen months after stoma placement, she was referred to our gynecology department for a second opinion. She had never been pregnant and had no abdominal pain or bowel obstruction symptoms when she presented to our hospital. The reason for seeking a second opinion was her desire for lesion resection and stoma closure. The stoma was placed on the right side of the upper abdomen. Blood laboratory findings were within normal limits except for CA125 (41 U/mL). Contrast enema revealed an obstruction from the sigmoid to the rectum (**Fig. 1**). Additionally, colonoscopy showed stenosis in the upper rectum, precluding the passage of the scope (**Fig. 2**). The oral side intestinal tract had no abnormality from the stoma. However, pelvic magnetic resonance imaging revealed a mass formation between the uterus and the rectum and adhesions from the uterus to the posterior vaginal vault (**Fig. 3**). A right ovarian cyst and a uterine fibroid were also observed. On the basis of these findings, after consultation with the gynecologist, we administered GnRH therapy for 4 months preoperatively and decided to perform surgery (laparoscopic low anterior resection, a Douglas fossa antecubital resection, and right ovarian cyst cauterization) for rectal obstruction caused by intestinal endometriosis.

**Fig. 4:**
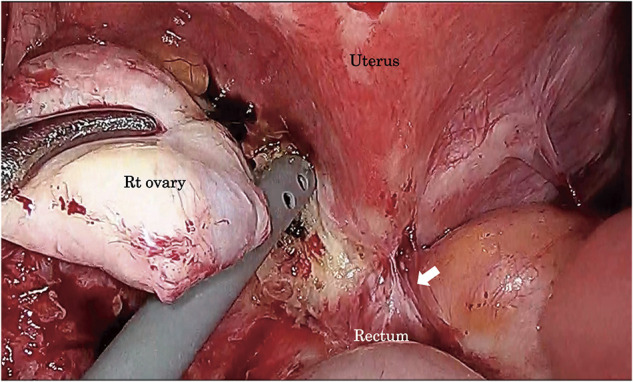
Severe fibrosis was observed in the uterus and the anterior rectum (arrow).

**Fig. 5:**
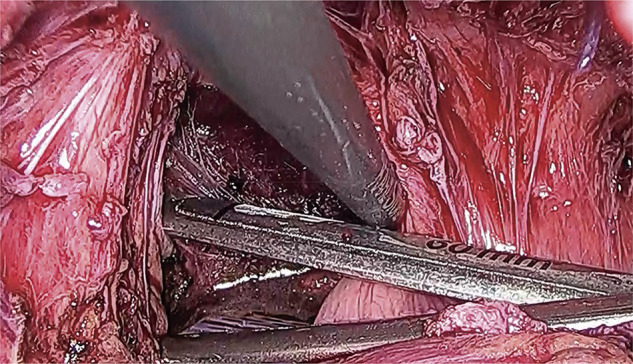
The lower rectum, which showed no wall changes was dissected.

**Fig. 6:**
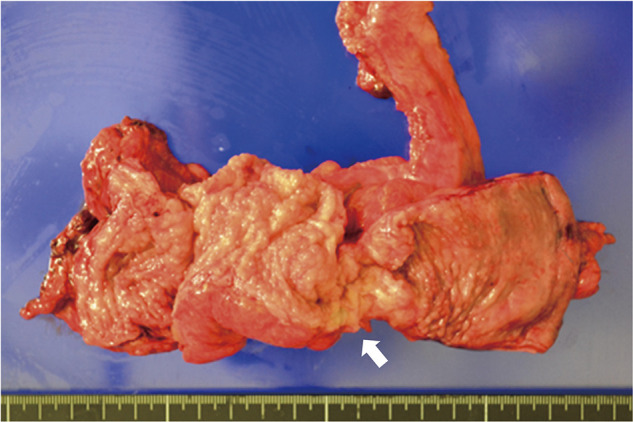
Bowel stenosis was observed at the site of wall thickening (arrow).

**Fig. 7:**
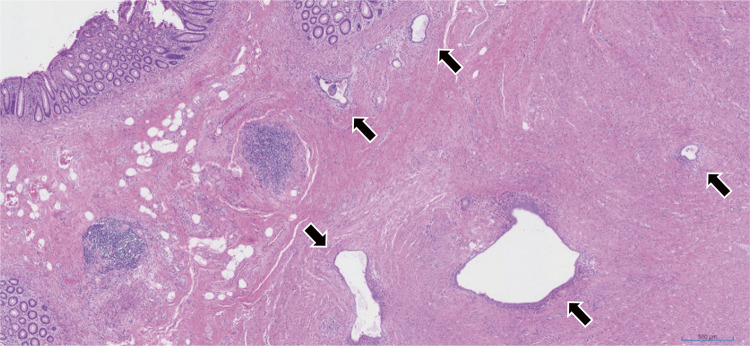
Endometrial glands (arrow) and stroma were found from the submucosa to the serosa, accompanied by fibrosis. There are no malignant findings.

After general anesthesia, stents were inserted into the bilateral ureters. Following insufflation, surgery was started with five ports. The abdominal cavity showed no adhesions other than the pelvis. Owing to the presence of a stoma in the transverse colon, avoiding the small intestine and obtaining a view into the pelvic cavity became difficult. There was localized thickening and induration of the intestinal wall, dark reddish-brown lesions on the serosal surface of the intestine, dense adhesions and a frozen pelvis, as well as fibrous adhesions to the posterior wall of the uterus. The space between the posterior uterus and the rectum was tightly adherent because of the endometriosis (**Fig. 4**). After inserting the uterine manipulator, the gynecologist aspirated, cauterized, and sutured laparoscopically the chocolate cyst of the right ovarian cyst. The gynecology surgery team was then replaced with the gastrointestinal surgery team. Identified in the sigmoid mesocolon area, the left ureter was carefully dissected into the pelvis, avoiding ureteral damage. The splenic flexure of the transverse colon was mobilized. After noticing that the serosal side of the rectum was thickened, the surgeon dissected the rectum from the peritoneal inversion to the anorectal side. The lower rectum, which demonstrated no wall change, was sliced using an Endo-GIA60 (Covidien, Minneapolis, MN, USA) (**Fig. 5**). Moreover, the umbilicus was incised by 5 cm. After blood flow evaluation by indocyanine green fluorescence, the intestine was cut at a sufficient distance from the rectal obstruction site, 6 cm on the oral side and 4 cm on the anal side (**Fig. 6**). Next, anastomosis with the DST was performed using DST Series EEA (Covidien). The endoscopic leak test was negative. The anastomosis was 7 cm from the anal verge. Histopathologically, the endometrial glands and stroma were found in the submucosa to the serosa, accompanied with fibrosis. None of them was malignant (**Fig. 7**). The patient had a favorable postoperative course, leading to her discharge on day 15. During her follow-up, intestinal obstruction due to recurrent intestinal endometriosis was not observed. After confirming the absence of stenosis in the anastomosis and the anorectal intestinal tract below the stoma through outpatient colonoscopy, the colostomy was closed approximately 12 months postoperatively. The patient has remained in a stable condition without any symptoms related to intestinal endometriosis.

## DISCUSSION

Endometriosis is a benign disease characterized by the ectopic proliferation of endometrial tissue. Endometriosis affects approximately 5%–10% of women of reproductive age. Macafee et al.^5)^ reported that the incidence of intestinal endometriosis is 12% among all endometriosis cases. Intestinal endometriosis is the most common form of ectopic endometriosis in women of sexual maturity, with its frequency increasing in recent years. Cases of intestinal occlusion due to endometriosis foci on the small or large bowel are even rarer, with a reported prevalence of 0.1%–0.7%.^6,7)^ The sigmoid colon and rectum were the most common sites of occurrence, accounting for approximately 70%–90%, followed by the small intestine in 7%, and the cecum in 3.6%.^8)^

Abdominal pain and bleeding are the most common complaints in intestinal obstruction resulting from intestinal endometriosis. Approximately half of the cases are related to the menstrual cycle. Preoperative imaging studies suggest that intestinal endometriosis diagnosis is made in only 37.8%–42% of cases, including suspected cases.^9,10)^

Endoscopically, intestinal endometriosis tends to take the form of a submucosal tumor and presents as an extraintestinal induration. Rarely, the mucosal surface may show erythema, hemorrhage, and erosion. The diagnostic rate of biopsy is approximately less than 6%, but recently, the tissue diagnostic rate of biopsy using endoscopic ultrasound-guided fine-needle aspiration is as high as 40%.^11)^ Our patient did not undergo biopsy. The diagnostic rate on biopsy was low, and although the clinical course was diagnostic of endometriosis, a biopsy should have been performed.

Treatment for intestinal endometriosis is aimed at improving the quality of life by alleviating symptoms and treating infertility. Considering most of the patients present in their twenties to forties, fertility preservation and social background should be considered when selecting treatment. Pharmacotherapy is the first-line treatment for intestinal endometriosis, but in cases of pain, infertility, difficulty in control by pharmacotherapy, or symptom worsening, surgical treatment may be indicated.^9)^ Improvement with pharmacotherapy alone has been reported, although such cases are extremely rare.^12)^ However, in general, the stenosis caused by intestinal endometriosis is considered irreversible, and surgical intervention is typically required.

In our case, wherein secondary changes such as fibrosis or lesion scarring are the cause of intestinal stenosis, gastrointestinal symptom improvement is unlikely, and surgical treatment should be considered. Additionally, preoperative pharmacotherapy may contribute to reducing intraoperative bleeding.^13)^

In our patient’s case, the lesion had adhesions to the fallopian tubes and ovaries. The patient was then treated with gentle and careful dissection to avoid damage to the ovaries and fallopian tubes for fertility preservation, which was possible in this case. Especially in the case of rectal endometriosis with deep endometriosis of the Douglas fossa, we placed ureteral catheters on both sides to avoid ureteral injury. We identified the left ureter in the sigmoid mesocolon, where fibrosis was relatively milder than that in the pelvis, and dissected it into the pelvis without damaging it. There was obliteration of the pouch of Douglas due to endometriosis, and elevation and fixation of the uterus using a uterine manipulator was useful in adhesiolysis. Compared with the conventional procedure, the laparoscopic procedure can be performed while looking at a magnified field, allowing clear visualization of the pelvic anatomy; therefore, the visible endometriosis can be easily identified and excised. Although the dissection took time, through the laparoscopic approach, the operation could be performed safely without causing damage to other organs.

**Fig. 8:**
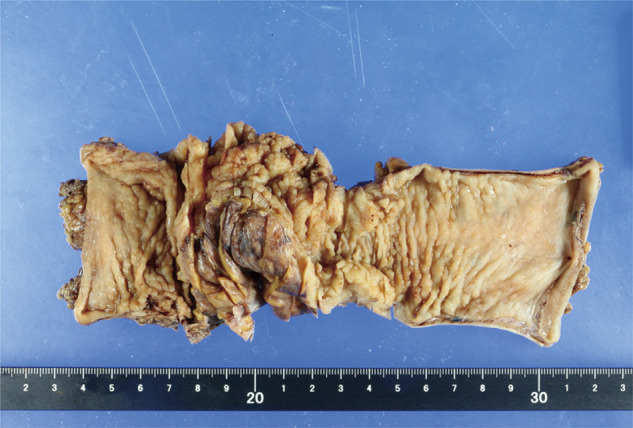
No macroscopic abnormalities were observed on the mucosal surface of the anal resection margin.

**Fig. 9:**
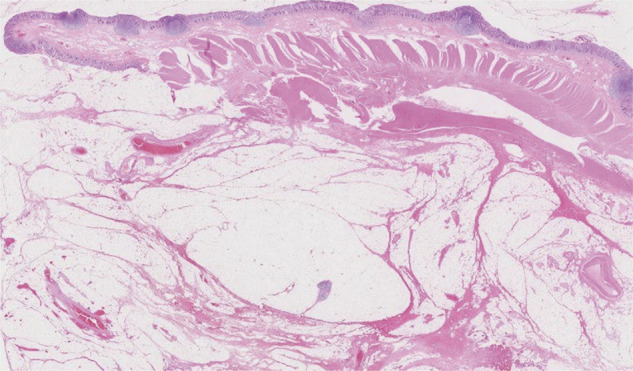
The anal side resection margin revealed no ectopic endometrial tissue.

In determining the extent of resection, the actual line of distal bowel resection tends to be lower than estimated because the extent of serosal endometrial changes preoperatively is difficult to be identified.^14,15)^ Preoperatively, a stenosis site from the sigmoid to the upper rectum was noted, but considering the spread of intestinal endometriosis, the lower rectum was cut below the peritoneal inversion. Regarding the distal resection margin, Kavallaris et al.^16)^ reported a recommended distance of 2 cm. Jinushi^15)^ et al. examined the presence of endometriotic foci at 2 cm intervals and found that such foci were frequently located at the 2 and 4 cm marks. Furthermore, it also reported that endometriotic foci were more frequently observed in areas with inflammatory thickening of the bowel wall.^15)^ In our case, the rectum was transected approximately 4 cm distal to the main lesion, at a site where no inflammatory thickening was observed on the serosal surface. This length may have contributed to the reduction of intestinal endometriosis recurrence after surgery (**Figs. 8** and **9**). The colon was extensively resected; hence, the colon at the splenic flexure needed to be transferred. Given the possibility of recurrence if endometrial tissue remains at the resection margin, careful judgment is required in dissection and resection, especially in rectal lesions with a frozen pelvis. It is stated that intestinal endometriosis spreads ectopically and is not a continuous lesion, making complete resection difficult.^15)^ Our policy is to perform resection at a sufficient distance from the stenotic lesion and at a site where there is no obvious wall thickening on the serosal surface. If wall thickening is observed at the resection margin, we are considering whether to perform frozen section analysis in future cases. Regarding rectal resection, the frozen pelvis was released and dissected down to the lower rectum, allowing safe resection at an area with little fibrotic change. The extent of the serosal lesions caused by endometriosis is difficult to diagnose preoperatively; thus, the extent of the lesion should be carefully diagnosed according to the intraoperative findings.

However, complications such as bladder injury, ureteral injury, rectovaginal fistula, suture failure, and pelvic abscess reportedly occur in approximately 10% of patients undergoing laparoscopic surgery.^17)^ Therefore, depending on the intraabdominal findings, open surgery must be performed to ensure a safe operation. By collaborating with gynecologists and urologists, we were able to perform laparoscopic surgery safely. Hence, laparoscopic surgery for intestinal endometriosis is a useful minimally invasive surgical technique.

## CONCLUSIONS

This report discusses a case of intestinal endometriosis resected under laparoscopic surgery. Although intestinal endometriosis is a good indication for laparoscopic surgery, a skilled endoscopic surgeon should perform the surgery because it requires a thorough knowledge of the anatomy and advanced laparoscopic techniques. In intestinal endometriosis, the extent of bowel resection is difficult to determine. Thus, the serosal changes in the endometriotic bowel should be monitored intraoperatively, and the distance from the site of wall thickening or bowel obstruction should be sufficient.

## DECLARATIONS

### Funding

The authors received no specific funding for this work.

### Authors’ contributions

HR and MKi performed the surgical procedures.

HR and KSa performed data interpretation and preparation of the manuscript.

YT and KSa compiled the surgical procedures.

YT, RT, KH, Masaya Kawai, SI, Kiichi Sugimoto, MT, HS, and TY contributed to the discussion.

All authors read and approved the final manuscript.

All authors agree to be responsible for all aspects of the study.

### Availability of data and materials

Data will be made available on reasonable request.

### Ethics approval and consent to participate

This report has been performed in accordance with the Declaration of Helsinki and was approved by the local ethics committee and conducted according to the guidelines of Juntendo University.

This work does not require ethical considerations or approval.

This study was conducted with the consent of the patients.

### Consent for publication

Informed consent to publish has been obtained from the patient.

### Competing interests

The authors of this manuscript have no conflicts of interest to disclose described by the *Surgical case reports*.
